# Investigation of colorectal cancer in accordance with consensus molecular subtype classification

**DOI:** 10.1002/ags3.12362

**Published:** 2020-07-21

**Authors:** Hiroshi Sawayama, Yuji Miyamoto, Katsuhiro Ogawa, Naoya Yoshida, Hideo Baba

**Affiliations:** ^1^ Department of Gastroenterological Surgery Graduate School of Medical Sciences Kumamoto University Honjo Japan

**Keywords:** colorectal cancer, consensus molecular subtype, prognostic marker

## Abstract

The classification of colorectal cancer (CRC) plays a pivotal role in predicting a patient's prognosis and determining treatment strategies. The consensus molecular subtype (CMS) classification system was constructed by analyzing genetic information from 18 CRC data sets, containing 4151 CRC samples. CRC was classified into four subtypes with distinct molecular and biological characteristics: CMS1 (microsatellite instability immune), CMS2 (canonical), CMS3 (metabolic), and CMS4 (mesenchymal). Since their designation in 2015, these classifications have been applied to basic and translational research of CRC, with the hope that understanding these subsets will influence a clinician's approach to therapeutic treatment and improve clinical outcomes. We reviewed CRC investigations in accordance with CMSs published in the last 5 years to further explore the clinical significance of these subtypes and identify underlying trends that may direct relevant future research. We determined that CMSs linked common features of CRC cell lines and PDX models in various studies. Furthermore, associations between prognosis and clinicopathological findings, including pathological grade and the stage of carcinogenesis, tumor budding, and tumor location, were correlated with CMS classification. Novel prognostic factors were identified, and the relationship between chemotherapeutic drug resistance and CMS has been fortified by our compilation of research; thus, indicating that this review provides advanced insight into clinical questions and treatment strategies for CRC.

## Graphical abstract

CRC was classified into four subtypes with distinct molecular and biological characteristics: CMS1 (microsatellite instability immune), CMS2 (canonical), CMS3 (metabolic), and CMS4 (mesenchymal). Novel prognostic factors were identified, and the relationship between chemotherapeutic drug resistance and CMS has been fortified by our compilation of research.

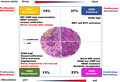

## 1 INTRODUCTION

**Figure 1:**
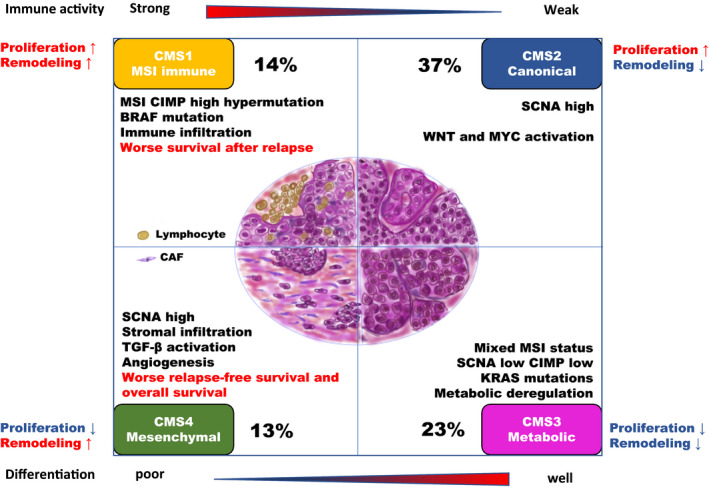
CMS classification. CMS1 (MSI immune 14%) was characterized by MSI, CIMP high hypermutation, BRAF mutation, immune infiltration and worse survival after relapse. CMS2 (Canonical, 37%) was characterized by SCNA high and WNT and MYC activation. CMS3 (Metabolic, 23%) was characterized by Mixed MSI status, SCNA low, CIMP low, KRAS mutations and metabolic deregulation. CMS4 (Mesenchymal, 13%) was characterized by SCNA high, stromal infiltration, TGF‐β activation, angiogenesis, worse relapse‐free survival and overall survival. MSI: microsatellite Instability, CIMP: CpG island methylator phenotype, BRAF: v‐raf murine sarcoma viral oncogene homolog B1, SCNA: somatic copy number alteration, KRAS: kirsten rat sarcoma viral oncogene homolog, TGF: transforming growth factor

Colorectal cancer (CRC) is the fourth leading cause of cancer‐related death worldwide,^1^ indicating a global need for better prognosis and treatment strategies. The TNM classification is commonly used to determine the progression of CRC; however, more in‐depth characterization is necessary to better assess treatment strategies and prognosis. Several classifications for CRC have been reported in accordance with gene signatures, the most robust of which is the consensus molecular subtype (CMS) system.^2^ CMS classification was devised from an analysis of 4151 CRC samples in 2015.^3^ This classification system consists of four subtypes (Figure 1); although tumor location is essential in determining current clinical practices, understanding the molecular characteristics and biological implications of CRC subsets helps to prevent an oversimplified approach to treatment.^4^ The genetic and pathological research demonstrated novel findings in accordance with the CMS classification. PubMed was explored for articles published in English with the terms “colorectal cancer” and “consensus molecular subtype.” In total, 103 articles were obtained, read, and analyzed for similar content and review article. Then, of these, 55 essential articles were selected for the references. This review was written for the medical doctors treating colorectal cancer and young researchers in the field of cancer research to gain more insights into genetic signatures of colorectal cancer. Cell lines and PDXs are fundamental elements for cancer research. Pathological findings and mutation status are essential for current medical practice. These findings have been partially explained with novel insights in accordance with CMS classification. Novel mechanism and prognostic factors have also been discovered by analyzing them in accordance with CMSs. This review takes a comprehensive look at CRC with regard to CMS classification, providing advanced insight that can be translated to clinical applications.

## 2 EXPLORING CMSS IN CELL LINES AND PATIENT‐DERIVED XENOGRAFTS

**Figure 2:**
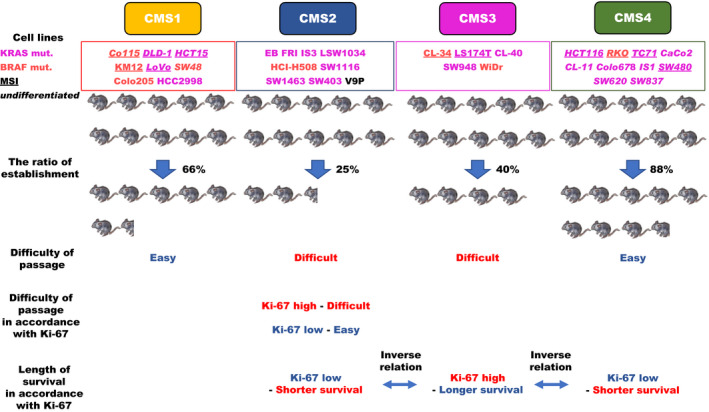
CMS classification of CRC Cell lines and PDXs. The KRAS, BRAF, MSI status and CMS classifications of CRC cell lines were shown. CMS2 PDXs with high expression of Ki‐67 grew slowly and were difficult to establish in comparison to PDXs with low expression of Ki‐67. CMS2 and CMS4 PDXs with low expression of Ki‐67 had shorter survival, whereas high expression of Ki‐67 in CMS3 PDXs resulted in shorter survival. KRAS: kirsten rat sarcoma viral oncogene homolog, BRAF: v‐raf murine sarcoma viral oncogene homolog B1, mut.: mutation, MSI: microsatellite Instability

Cell lines and patient‐derived xenograft (PDX) models are commonly used in basic and translational research.^5^ The DNA, RNA, and protein profiling of 34 CRC cell lines were examined and subsequently classified into the CMSs.^6^ In an investigation using PDXs, the success of establishing PDXs of CMS1, CMS2, CMS3, and CMS4 were 66%, 25%, 40%, and 88%, respectively. Passages of PDXs were difficult in the CMS2 and CMS3 conditions, and the enrichment of CMS1 and CMS4 were detected in later passages. The Ki‐67 expression was associated with both establishment and survival of PDXs^7^ (Figure 2). Importantly, mesenchymal tumors, CMS4, can be passaged, but not maintain their features or subtype. Several stromal cells or human tumors decreased and were replaced by murine counterparts in subsequent passages.^8^ Stromal‐derived gene expression is absent in these human‐specific gene expression profiles. The Colorectal Cancer Intrinsic Subtypes (CRIS) classification system was developed by transcriptional profiling from large PDXs collection with human‐specific prove. This approach allows an assessment of gene expression originating only from the cancer cells. The CRIS consisted of five categories as follows: CRIS‐A, characterized by mucinous, glycolytic, MSI or KRAS mutation; CRIS‐B, TGF‐β activation and EMT; CRIS‐C, elevated EGFR signaling; CRIS‐D, WNT activation and IGF2 amplification; and CRIS‐E, Paneth cell‐like phenotype and TP53 mutation. CRIS subtypes successfully stratify independent sets of primary and metastatic CRCs by minimizing the confounding effects of stromal‐derived intratumoral heterogeneity.^9^

## 3 PREDICTING CMS BY IMMUNOHISTOCHEMISTRY, MRNA, AND MIRNA

Whole genome sequencing was essential to the classification of CMSs; however, the cost of this procedure prevents it from being used routinely in a clinical setting. Various studies have focused on the use of other methods, such as immunohistochemistry to determine CMS. Trinh et al determined the microsatellite instability (MSI) status of their samples and designated patients with high MSI to the CMS1 category. Using immunohistochemistry, the remaining patients were classified into "epithelial" CMS2/3 or "mesenchymal" CMS4 subtypes based on four markers: CDX2, FRMD6, HTR2B, and ZEB1. This method demonstrated 87% concordance compared with the transcriptome‐based classification.^10^, ^11^

CMS4 has been associated with drug resistance and poor prognosis compared with the other CMSs, making it the primary focus of many studies. The expression of PDGFRA, PDGFRB, PDGFC, and KIT mRNA were found to be predictive of CMS4, with an area under the curve of 0.95, and 95% confidence interval 0.94‐0.97.^12^

## 4 THE ASSOCIATION OF CLINICOPATHOLOGICAL FINDINGS AND CMSS

Mucinous histology and budding score are associated with poor prognosis of CRC patients; one study indicated that 277 of the 1877 (14.8%) CRC patients tested were positive for mucinous histology. Mucinous CRC was classified into CMSs: CMS1　(34.0%), CMS2 (6.4%), CMS3 (29.8%), and CMS4 (29.8%), and CMS2, the major CRC subtype, represented the smallest proportion of cases with mucinous histology. The SMAD4, GNAS, ERBB2, BRAF, and KRAS mutations occurred at higher frequencies in mucinous types, while TP53, APC, and NRAS mutations were less common.^13^

Tumor budding was investigated within the four cohorts. High budding (≥5 buds) was preferentially classified as CMS4; CRC patients with high budding had an unfavorable prognosis in those cohorts.^14^ Molecular subtype may be switching from CMS2 to CMS4 in the budding regions as seven of the eight samples were classified as CMS2 at the tumor center, yet five of these samples closely matched CMS4 at the budding region.^15^ A limitation of classifying CRC by CMS is that intratumor heterogeneity is often detected in biopsy samples. The CMS classification using biopsy samples is significantly less reliable, with 43% of cases unknown in biopsy vs 13% unknown in resections.^16^ Further investigation is necessary to determine how this information can help to inform decisions in a clinical setting.

## 5 CARCINOGENESIS, CANCER PROGRESSION, AND METASTASIS IN ACCORDANCE WITH CMSS

Recent investigations have revealed the characteristic genetic changes associated with adenoma, primary lesions, and metastatic lesions during cancer progression. In one study, sporadic adenoma polyps (n = 311) were classified into their CMSs: CMS1 (21.9%), CMS2 (69.5%), CMS3 (5.1%), and CMS4 (1.6%). Interestingly, most adenomatous polyps were classified as CMS2, whereas 57.1% of hyperplasic polyps and 76.5% of serrated adenomas were identified as CMS1. CMS1 polyps are more frequently presented in right‐sided colon whereas CMS2 polyps are more frequently presented in left‐sided colon.^17^ In another study, 51 lesions were divided into high risk adenoma (n = 13) and low risk adenoma (n = 39); 67% of CMS2 cases were designated as high‐risk and 82% of low‐risk adenoma was classified into CMS3.^18^ CMS2 sporadic adenoma polyps may transition to CMS1 CRC via MMR deficiency and increased DNA damage. Additionally, CMS2 adenoma may become CMS3 through the mutation of KRAS, activation of the MAPK pathway and metabolic degradation, and may transition to CMS4 through TGF‐β activation.^17^ While a CMS4‐like phenotype was rarely represented in adenoma, sessile serrated adenoma/polyp acquired an epithelial to mesenchymal (EMT)‐like phenotype by TGF‐β activation in early stage polyps.^19^

**Figure 3:**
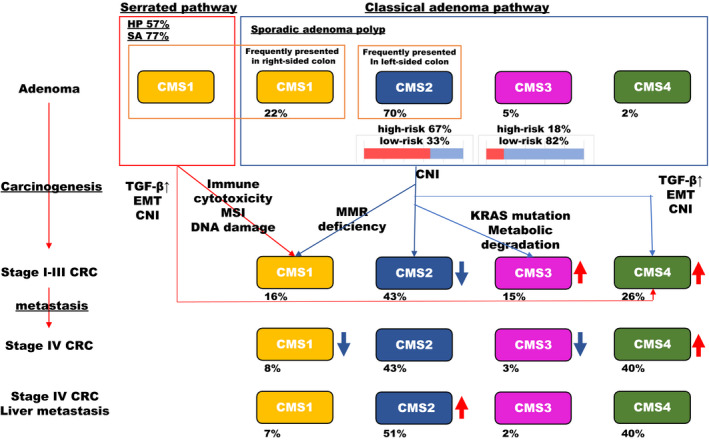
CMS classification in carcinogenesis cancer progression and metastasis. The characteristic genetic and the proportion of CMS classification changes associated with adenoma, primary lesions, and metastatic lesions during cancer progression

Fontana et al reported the proportions of each CMS at various stages of carcinogenic progression. CRC cases without distant metastasis at diagnosis (n = 2715) were classified: CMS1, 16%; CMS2, 43%; CMS3, 15%; and CMS4, 26%. The patients with distant metastasis at diagnosis (n = 236) were distributed accordingly: CMS1, 8%; CMS2, 43%; CMS3, 9%; and CMS4, 40%. Liver metastasis cases (n = 57) were divided as follows: CMS1, 7%; CMS2, 51%; CMS3, 2%; and CMS4, 40%.^20^ Another cohort also demonstrated that very few cases classified as CMS3 represent metastatic lesions (<1%) compared to primary lesions, of which CMS3 comprises 11%.^21^ These findings indicated that CMS3 may become uncommon as CRC progresses (Figure 3).

Interestingly, intra‐patient heterogeneity between primary lesions and peritoneal metastatic lesions was frequently observed in a cohort of 28 patients, three‐quarters of which were diagnosed with CMS4 peritoneal carcinomatosis. Fifteen of the 16 patients with paired tumors, a primary lesion and one to four metastatic lesions, had at least one CMS4‐positive tumor.^22^

## 6 PROGNOSIS IN ACCORDANCE WITH CMSS

**Figure 4:**
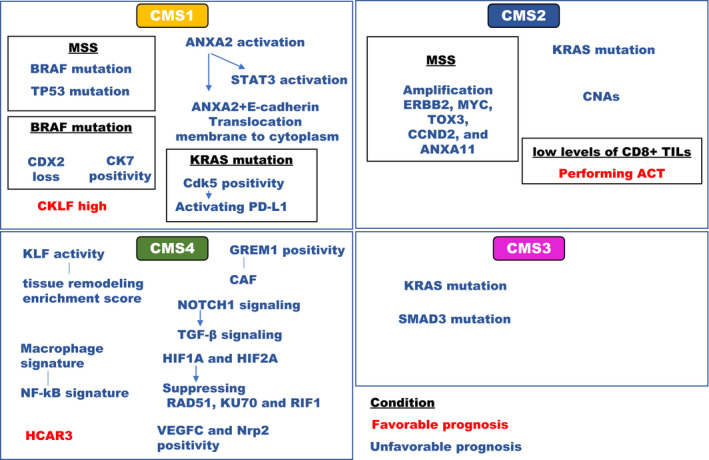
Prognostic factors in accordance with CMSs. Several prognostic genes were discovered in investigations that focused on CMS classification. MSS: microsatellite stable, MSI: microsatellite Instability, CDX2: caudal‐type homeobox 2, CK7: cytokeratin 7, ANXA2: annexin A2, CKLF: chemokine like factor, STAT3: signal transducer and activator of transcription 3, Cdk5: cyclin‐dependent kinase 5, PD‐L1: programmed cell death ligand 1, ERBB2: erb‐b2 receptor tyrosine kinase 2, TOX3: thymocyte selection associated high mobility group box 3, CCND2: cyclin D2, ANXA11: annexin A11, CNA: copy number alteration, TILs: tumor infiltrating lymphocytes, KLF: kruppel‐like factor, CLDN2: claudin 2, NF‐kB: nuclear factor kappa B, GREM1: gremlin 1, CAF: cancer‐associated fibroblast, TGF‐β: transforming growth factor beta‐1, HIF: hypoxia inducible factor, RIF1: replication timing regulatory factor 1, VEGFC: vascular endothelial growth factor C, NRP2: neuropilin 2, HCAR3: hydroxycarboxylic acid receptor 3

Many prognostic factors were not associated with the CMS classification. NUSAP1, CD44, and COL4A1 have been detected among all CMSs and play a key role in CRC progression. NUSAP1 regulates BRCA1 protein levels, CD44 presents as an EMT marker, and COL4A1 is a tumor angiogenesis indicator.^23^ Though many markers have been identified and subsequently associated with CMSs, several prognostic genes were discovered in studies that focused on CMS classification. We reviewed prognostic genes in accordance with CMSs as follows (Table 1 and Figure 4).

**Table 1:** Prognosis in accordance with CMS classification

CSM	Status	Factor	Reference		HR	95%CI	*P* value	Ref.
CMS1	MSS	BRAF mut.	BRAF WT	OS	7.73	2.35‐255.4	.001*	[24]
	MSI	BRAF mut.	BRAF WT	OS	1.05	0.44‐2.50	.912	
CMS1	BRAF mut.	CDX2 loss	CDX2 normal	OS	1.72	1.03‐2.86	.036*	[25]
		CK7 positive	CK7 negative	OS	2.17	1.10‐4.29	.026*	[26]
	MSS	TP53 mut.	TP53 WT	OS	5.52	1.21‐25.3	.013*	[30]
	MSI	TP53 mut.	TP53 WT	OS	0.68	0.15‐3.01	.610	
CMS1	KRAS WT	Cdk5 high	Cdk5 low	DFS	1.32	0.40‐4.35	.740	[31]
	KRAS mut.	Cdk5 high	Cdk5 low	DFS	7.53	1.56‐36.46	.012*	
CMS1		CKLF high	CKLF low	RFS	0.21	0.04‐0.89	NA*	[32]
CMA1‐4		BRAF class1 mut.	BRAF class1 WT	OS	2.38	1.61‐3.54	NA*	[30]
CMS2/3		BRAF class2 mut.	BRAF class2 WT	OS	1.90	0.85‐4.26	NA	
		BRAF class3 mut.	BRAF class3 WT	OS	1.90	0.51‐1.69	NA	
CMS1/4		KRAS mut.	KRAS WT	OS	0.97	0.61‐1.52	.880	[24]
CMS2/3		KRAS mut.	KRAS WT	OS	1.73	1.19‐2.50	.004*	
CMS2	Stage I‐III MSS	High amplif. (CNAs)	Low amplif. (CNAs)	OS	3.20	1.30‐7.90	.010*	[34]
CMS2	CRIS‐C Stage III	ACT	Surgery alone	OS	0.11	0.01‐0.81	.030*	[35]
	CRIS‐C Stage II	ACT	Surgery alone	OS	0.15	0.06‐0.42	<.001*	
CMS3		SMAD4 mut.	SMAD4 WT	OS	2.08	1.50‐2.88	<.001*	[36]
CMS/1/3	Stage III	BCL‐2		OS	5.20	1.4‐1.79	.020*	[37]
CMS4		HCARS module		OS	2.09	1.29‐3.39	.003*	[23]

Abbreviations

ACT: adjuvant chemotherapy; amplif.: amplification; BRAF: v‐raf murine sarcoma viral oncogene homolog B1; Cdk5: cyclin‐dependent kinase 5; CDX2: caudal‐type homeobox 2; CI: confidential interval; CK7: cytokeratin 7; CKLF: chemokine like factor; CNA: copy number alteration; DFS: disease‐free survival; HR: hazard ratio; KRAS: kirsten rat sarcoma viral oncogene homolog; MSI: microsatellite Instability; MSS: microsatellite stable; mut.: mutation; OS: overall survival; Ref: reference; RFS relapse‐free survival; WT: wild type.

* Significant difference.

### 6.1 Prognostic factors in CMS1

In CMS1, microsatellite stable (MSS) CRC patients with BRAF mutation are associated with a shorter overall survival (OS) compared with BRAF wild type; however, prognostic difference between BRAF mutation or BRAF wild type were not found in MSI CRC patients.^24^ In the BRAF mutated metastatic CRC patients, CDX2 loss and CK7 positivity indicated unfavorable prognosis.^25^, ^26^ CpG island methylator phenotype (CIMP) can result in the silencing of key genes important for tumor progression, including the tumor‐suppressor gene, CDKN2A, and the DNA mismatch repair gene, MLH1. The CIMP‐H1 cluster was enriched for cancers with features characteristic of serrated tumors and those containing a BRAF mutation.^27^

Loss‐of‐function mutations of JAK1 are found in 20% of CRCs. These tumors show elevated transcriptional signatures that are associated with resistance to anti‐programmed death‐1 treatment. Among the MSI tumors, the total mutation load correlated with the number of predicted neoantigens, but not with immune cell infiltration, which was dependent on the CMS. CMS1 in particular had higher immunogenic features compared with CMS2‐4.^28^ Additionally, the expression of Annexin A2 (ANXA2), which is associated with endocytic and exocytic events and cytoskeleton regulation, was elevated in CMS1‐classified CRCs. In cancer cells, TGF‐β stimulation increased ANXA2 expression and phosphorylation, and phosphorylated ANXA2 activated the STAT3 pathway, resulting in EMT and invasion.^29^

TP53 mutations are found in 60% of CRCs. TP53 mutations have subtype‐dependent associations with metastatic propensity and patient prognosis, potentially mediated by a CMS1‐specific immunomodulatory effect. Specifically, TP53 mutant CMS1 CRC cases with MSS were associated with poor prognosis.^30^ Cyclin‐dependent kinase 5 (Cdk5) is associated with migration and is lowly expressed in CMS1 compared with the other CMSs. High Ckd5 expression in CMS1 was associated with a shorter progression free survival (PFS), but not in the other CMSs. Ckd5 is associated with the upregulation of IFN‐induced programmed death ligand‐1, indicating cancer immunoediting.^31^ Additionally, CKLF expression, which is linked to lymphocyte infiltration, was associated with favorable prognosis in CMS1 with MSI.^32^

### 6.2 Prognostic factors in CMS2

RAF and RAS status has differing implications for prognosis on the basis of CMS classification. BRAF mutations are classified into the three groups: class 1‐V600E, class 2‐codons 597/601, and class 3–codons 594/596. The 117 patients with BRAF mutations were stratified into Class 1 (n = 92), Class 2 (n = 12), and Class 3 (n = 13). Class 2 and 3 patients were more likely to belong to CMS 2/3. Class 1 patients had a shorter OS compared with BRAF wild‐type; however, Class 2/3 CRC patients were not different from BRAF controls with respect to OS.^33^ Survival of CMS1/4 patients is not affected by KRAS status; however, the CMS2/3 patients with KRAS mutations have a shorter OS than those with wildtype KRAS.^24^

Copy number driven gene expression was enriched for pathways characteristic of CMS2, including DNA repair and cell cycle progression. The gene expression in CMS2 CRCs is driven by CNAs to a much larger extent than in the other CMSs. The copy number‐related genetic basis was heavily influenced by gene expression signals from the tumor microenvironment in CMS2. ERBB2, MYC, TOX3, CCND2, and ANXA11 indicating that high‐frequency focal amplification were associated with a poor survival among patients with stage I‐III MSS CRCs.^34^

CRIS‐C patients displayed low levels of CD8+ tumor‐infiltrating lymphocytes (TILs), and, notably, 50.2% of the CMS2 CRC patients were divided into CRIS‐C, and the CRIS‐C patients in CMS2 had favorable prognosis with adjuvant chemotherapy (ACT) compared with surgery alone, in stage II and III.^35^

### 6.3 Prognostic factors in CMS3

Fewer articles have focused on CMS3 compared with the other CMSs. One study indicated a relationship between CMS3 and the presence of a SMAD4 mutation. In this cohort, 12% of CRC patients had a SMAD4 mutation; these patients had a shorter OS compared with wild‐type SMAD4 patients. SMAD4 mutations frequently occurred with KRAS, NRAS, and BRAF mutations, and were more common in CMS3.^36^ The stage III CRC patients with high risk as classified by a mathematical model of BCL‐2 protein interactions had a shorter OS compared with low risk patients. Additionally, BCL‐2‐dependent signaling resulted in resistance to chemotherapy in CMS1 and CMS3.^37^

### 6.4 Prognostic factors in CMS4

The CRCs have been further characterized into two gene signatures on the basis of cell proliferation and tissue remodeling. The CRC patients whose gene signature indicated cell proliferation lead to a favorable prognosis, while indication of cell remodeling lead to an unfavorable prognosis. CRC with both a remodeling and less proliferative signature (74% of CMS4) had the poorest survival (Figure 1). KLF4 is a transcription factor, involved with tissue remodeling, and has been associated with poor prognosis in CRC.^38^

Gremlin1 (GREM1) expression is significantly higher in CRC CMS4 compared to the other CMSs. GREM1 was associated with levels of cancer‐associated fibroblasts in the tumor microenvironment.^39^ Activation of NOTCH1 signaling in the murine intestinal epithelium leads to highly penetrant metastasis in KRAS‐driven serrated cancers via neutrophil specific TGF‐β signaling.^40^ A macrophage signature was strongly associated with the cancer‐associated fibroblast signature in large cohorts. Macrophage positivity was associated with unfavorable prognosis and was identified in CMS4. Additionally, an M2 macrophage activated NF‐κB signature was present in CMS1/4, and M2 macrophages induced loss of TJ proteins at regions of tumor cell‐cell contact.^41^

DNA repair was inversely correlated with hypoxia‐inducible factor (HIF) 1A and HIF2A, which were strongly suppressed in CMS4. High expression of HIF1A and low expression of the repair proteins RAD51, KU70, and RIF1 was significantly associated with unfavorable prognosis.^42^ Lymphangiogenic gene expression was associated with poor prognosis in both primary and liver metastasis of CRC. Lymph node recurrence following CRC liver metastasis resection was associated with high expression of VEGFC and Nrp‐2. VEGFC and Nrp‐2 expression was elevated in CMS4 and these genes were associated with poor prognosis in CMS4.^43^ The HCAR3 module was associated with favorable prognosis in CMS4. HCAR3 acts as a tumor suppressor and has been implicated in multiple interactions as well as in the development of anti‐cancer drugs.^23^

## 7 SENSITIVITY TO CYTOTOXIC DRUGS IN ACCORDANCE WITH CMSS

**Figure 5:**
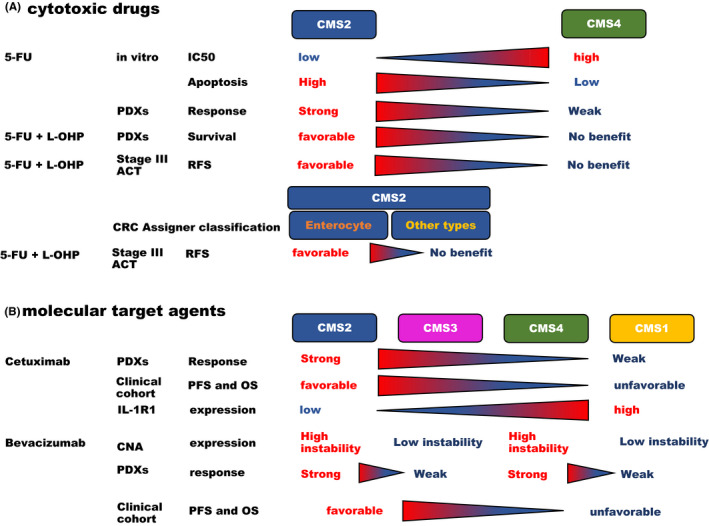
Therapeutic effects of cytotoxic drugs and molecular target agents in accordance with CMS. Therapeutic effects of cytotoxic drugs were shown. CMS4 had a poor response to 5‐FU compared with CMS2. CMS2 was a longer relapse‐free survival (RFS) compared with CMS4 (A). Therapeutic effects of molecular target agents were shown. CMS2 responded most significantly to anti‐epidermal growth factor receptor antibody, cetuximab, treatment compared with the other CMSs. CMS1 had the least significant response. CMS1/3 had a poor response to anti‐vascular endothelial growth factor, bevacizumab, compared with CMS2/4 (B). 5FU: fluorouracil, IC: inhibitory concentration, PDX: patient‐derived xenograft, ACT: adjuvant chemotherapy, PFS: progression free survival, OS: overall survival, IL‐1: interleukin‐1, CNA: copy number alteration

The effects of anti‐cancer drugs differ according to CMS classification (Table 2 and Figure 5). The sensitivity of CRC cell lines to the cytotoxic drugs, 5‐fluorouracil (5‐FU) and L‐OHP, was reported in accordance with CMSs. The inhibitory concentration 50 (IC50) of 5‐FU was lower to treat the CMS1‐3 cell lines compared with CMS4. The ratio of apoptotic cells in CMS2 was high after treating with 5‐FU combined with L‐OHP in comparison with CMS4. The CMS2 PDXs with 5‐FU and L‐OHP treatment resulted in longer survival than the placebo treatment; however, the survival of CMS4 PDXs showed no benefit with the combined treatment.^44^

**Table 2:** Therapeutic effects of cytotoxic drugs in accordance with CMSs

CMS	Status	Drugs	Factor	Reference		HR	95%CI	*P* value	Ref.
CMS2	Stage II	5‐FU based CT.	Performed	Not performed	OS	0.21	0.05‐0.90	.0035*	[35]
CMS2/3	Stage III	5‐FU based CT.	Performed	Not performed	OS	0.2	0.11‐0.38	<.001*	
CMS2	Stage II CRIS‐C	5‐FU based CT.	Performed	Not performed	OS	0.11	0.01‐0.81	.03*	
CMS2	Stage II CRIS‐C	5‐FU based CT.	Performed	Not performed	OS	0.15	0.06‐0.42	<.001*	
CMS1	Stage III	5‐FU ± L‐OHP	5‐FU + L‐OHP	5FU alone	RFS	0.77	0.46‐1.29	.32	[47]
CMS2	Stage III	5‐FU ± L‐OHP	5‐FU + L‐OHP	5FU alone	RFS	0.61	0.43‐0.87	.006*	
CMS3	Stage III	5‐FU ± L‐OHP	5‐FU + L‐OHP	5FU alone	RFS	1.17	0.54‐2.53	.68	
CMS4	Stage III	5‐FU ± L‐OHP	5‐FU + L‐OHP	5FU alone	RFS	0.87	0.64‐1.19	.39	
CMS2	Stage III enterocyte	5‐FU ± L‐OHP	5‐FU + L‐OHP	5FU alone	RFS	0.2	0.07‐0.59	.003*	
CMS2	Stage III other	5‐FU ± L‐OHP	5‐FU + L‐OHP	5FU alone	RFS	0.77	0.50‐1.18	.24	
CMS4	mCRC	IRI or L‐OHP	IRI based CT.	L‐OHP based CT.	PFS	0.31	0.13‐0.64	NA*	[49]
CMS4	mCRC	IRI or L‐OHP	IRI based CT.	L‐OHP based CT.	OS	0.45	0.19‐0.99	NA*	

Abbreviations

5FU, fluorouracil; CI, confidential interval; CT, chemotherapy; HR, hazard ratio; IRI, irinotecan; mCRC, metastatic colorectal cancer; NA, non assessment; OS, overall survival; PFS, progression‐free survival; RFS, relapse ‐free survival.

* Significant difference.

In PDXs, CMS1/4 had a poor response to 5‐FU compared with CMS2/3.^45^, ^46^ In stage II and III, CRC patients classified as CMS1/4, no survival benefit was conferred by ACT. The prognosis of the stage II CRC CMS2 patients was improved by ACT, and the survival of the stage III CRC patients in CMS2/3 had a favorable prognosis in those receiving ACT compared to those who only received surgery. The stage II and III CRC CMS2 patients with CD8 positivity had a favorable prognosis without ACT.^35^ In a different cohort, the stage III CMS2 patients with 5‐FU and L‐OHP treatment had a more favorable prognosis than those receiving 5‐FU monotherapy. More specifically, only the enterocyte subtypes of CRC Assigner (CRCA) classification in CMS2 patients had a significant benefit from the 5‐FU plus L‐OHP treatment. The benefit of adding L‐OHP to 5‐FU was not shown in the other subtypes.^47^ ZEB2 positivity was found in CMS4 and was elevated after L‐OHP treatment. High ZEB2 expression correlated with reduced proliferation, however, ZEB2 positivity was also associated with resistance to chemotherapy and poor prognosis.^48^

Regarding first‐line chemotherapy, Irinotecan‐based regimens were significantly superior to L‐OHP‐based regimens for PFS in CMS4. TOP1 and CES2 expression are predictive biomarkers for response to irinotecan and these genes expression levels were significantly elevated in CMS4 in this cohort.^49^

## 8 SPECIFIC MOLECULAR TARGETED AGENTS AND CMSS

In an investigation with PDXs, CMS2 responded most significantly to the cetuximab treatment compared with the other CMSs, and CMS1 had the least significant response.^45^, ^46^ In a clinical cohort, investigating anti‐EGFR therapy, CMS1 particularly showed a shorter PFS and OS, and CMS2 showed a longer PFS and OS compared with the other CMSs.^49^ Interleukin‐1 (IL‐1) R1 mRNA levels were associated with the cetuximab treatment response. IL‐1R1 expression was elevated in the CMS1/4 compared with CMS2/3, and CMS1 CRC patients with high IL‐1R1 expression had a significantly shorter PFS than those with low IL‐1R1 expression.^50^

Smeets et al demonstrated that CNA profiles are associated with the benefit of the bevacizumab treatment. Clustering of CNA data from mCRC identified 3 CNA clusters. Cluster 1 tumors were characterized by a strong immune‐activated microenvironment, while cluster 2 and 3 tumors were characterized by angiogenesis, epithelial‐to‐mesenchymal transition, and inflammatory response pathway. An overlap between CMS subtypes and CNA clusters was also found. CMS1/3 tumor was likely to classify into CNA cluster 1 and CMS2/4 into CNA clusters 2 and 3. CMS2/4 showed additional benefit from the bevacizumab treatment combined with cytotoxic drugs compared with cytotoxic drugs alone. Hypermutator phenotypes, such as tumors with POLE or POLD1 mutations, or MSI tumors, showed no additional benefit with the bevacizumab treatment. Importantly, MSS tumors with a stable copy number profile showed no additional benefit from the bevacizumab treatment.^51^ In the AGITG MAX trial, patients with CMS2/3 showed benefits from the combination chemotherapy with bevacizumab compared with CMS1/4 in the first line chemotherapy.^52^.

CMS1 patients with bevacizumab treatment had favorable prognosis compared with those with cetuximab. In contrast, CMS2 patients with cetuximab treatment had favorable prognosis compared with those with bevacizumab treatment in CALBG/SWOG80405. KRAS mutant ratios of CMS1, CMS2, CMS3, and CMS4 were 69.2, 56.6, 94.1, and 70.7, respectively, in this study.^53^ Both anti‐EGFR antibody and anti‐VEGF antibody treatments offered fewer benefits for CMS1/4 compared with CMS2/3. In the FIRE3 trial, prognostic difference between anti‐EGFR or anti‐VEGF agents were not found in accordance with CMSs. CMSs did not impact selection of these anti‐molecular target agents in first‐line chemotherapy^54^, ^55^ (Table 3 and Figure 5).

**Table 3:** Therapeutic effects of molecular target agents in accordance with CMS

CMS	Status	Drugs	Factor	Reference		HR	95%CI	*P* value	Ref.
CMS1‐4	mCRC	anti‐EGFR antibody	CMS1	Other CMSs	PFS	2.50	1.31‐4.39	<.001*	[49]
CMS1‐4	mCRC	anti‐EGFR antibody	CMS1	Other CMSs	OS	4.23	1.83‐9.04	<.002*	
CMS1‐4	mCRC	anti‐EGFR antibody	CMS2	Other CMSs	PFS	0.67	0.44‐1.01	.05	
CMS1‐4	mCRC	anti‐EGFR antibody	CMS2	Other CMSs	OS	0.49	0.27‐0.87	.049*	
CMS1	mCRC	cetuximab	IL‐1R1 high	IL‐1R1 low	PFS	2.74	1.54‐4.87	<.001*	[50]
CMS2	mCRC	cetuximab	IL‐1R1 high	IL‐1R1 low	PFS	0.58	0.31‐1.09	.085	
CMS3	mCRC	cetuximab	IL‐1R1 high	IL‐1R1 low	PFS	1.44	0.83‐2.51	.19	
CMS4	mCRC	cetuximab	IL‐1R1 high	IL‐1R1 low	PFS	1.27	0.88‐1.85	.2	
	mCRC CIN‐high	BVZ	CT + BVZ	CT	PFS	0.70	0.54‐0.90	.006*	[51]
	mCRC CIN‐low	BVZ	CT + BVZ	CT	PFS	0.91	0.45‐1.84	.798*	
CMS1	mCRC	CT ± BVZ	CB + CBM	C	PFS	0.83	0.43‐1.62	.99	[52]
CMS2	mCRC	CT ± BVZ	CB + CBM	C	PFS	0.50	0.33‐0.76	<.001*	
CMS3	mCRC	CT ± BVZ	CB + CBM	C	PFS	0.31	0.13‐0.75	.04*	
CMS4	mCRC	CT ± BVZ	CB + CBM	C	PFS	1.24	0.68‐2.25	.32	
CMS1‐4	mCRC	CT ± BVZ	CB + CBM	C	PFS	0.67	0.50‐0.90	.008*	
CMS1	mCRC	BVZ or cetuximab	Cetuximab	BVZ	OS	2.34	1.48‐3.70	<.001*	[53]
CMS2	mCRC	BVZ or cetuximab	Cetuximab	BVZ	OS	0.62	0.45‐0.86	.0046*	
CMS3	mCRC	BVZ or cetuximab	Cetuximab	BVZ	OS	1.09	0.45‐1.94	.7606	
CMS4	mCRC	BVZ or cetuximab	Cetuximab	BVZ	OS	1.04	0.72‐1.51	.8336	
CMS1	mCRC	BVZ or cetuximab	Cetuximab	BVZ	PFS	2.28	1.47‐3.55	<.001*	
CMS2	mCRC	BVZ or cetuximab	Cetuximab	BVZ	PFS	0.91	0.68‐1.21	.5150	
CMS1	mCRC	BVZ or cetuximab	Cetuximab	BVZ	PFS	1.10	0.64‐1.88	.7395	
CMS2	mCRC	BVZ or cetuximab	Cetuximab	BVZ	PFS	0.87	0.62‐1.23	.4361	
CMS1	mCRC RAS WT	BVZ or cetuximab	Cetuximab	BVZ	PFS	1.05	0.57‐1.94	.87	[54]
CMS2	mCRC RAS WT	BVZ or cetuximab	Cetuximab	BVZ	PFS	1.04	0.73‐1.43	.82	
CMS3	mCRC RAS WT	BVZ or cetuximab	Cetuximab	BVZ	PFS	0.82	0.40‐1.70	.59	
CMS4	mCRC RAS WT	BVZ or cetuximab	Cetuximab	BVZ	PFS	0.67	0.45‐0.99	.0048*	
CMS1‐4	mCRC RAS WT	BVZ or cetuximab	Cetuximab	BVZ	PFS	0.91	0.72‐1.14	.41	
CMS1	mCRC RAS mut.	BVZ or cetuximab	Cetuximab	BVZ	PFS	1.89	0.61‐5.88	.28	
CMS2	mCRC RAS mut.	BVZ or cetuximab	Cetuximab	BVZ	PFS	1.28	0.62‐2.65	.51	
CMS3	mCRC RAS mut.	BVZ or cetuximab	Cetuximab	BVZ	PFS	1.08	0.51‐2.28	.84	
CMS4	mCRC RAS mut.	BVZ or cetuximab	Cetuximab	BVZ	PFS	1.37	0.75‐2.51	.31	
CMS1‐4	mCRC RAS mut.	BVZ or cetuximab	Cetuximab	BVZ	PFS	1.34	0.94‐1.93	.11	

Abbreviations

5 IL‐1, interleukin‐1; bevacizumab and mitomycin; BVZ, bevacizumab; C, capecitabine; CB, capecitabine plus bevacizumab; CBM capecitabine; CI, confidential interval; CIN, chromosomal instability; HR, hazard ratio; mCRC, metastatic colorectal cancer; mut., mutation; OS, overall survival; PFS, progression‐free survival; RAS, rat sarcoma viral oncogene homolog; Ref., reference; WT, wild type.

* Significant difference

## 9 FUTURE PERSPECTIVES AND SUMMARY

This review describes half a decade of development in CRC research on the basis of CMS classification. The CMS classification system has been impactful on our contemporary understanding of CRC with regard to carcinogenesis, cancer progression, and drug resistance. Here, we have explored how the CMS classifications can help explain the heterogeneity of CRC. Furthermore, novel prognostic factors, mechanisms of cancer progression, and therapeutic agents were evaluated through an in‐depth analysis of current literature, and how recent studies have linked these factors to various CMSs. CMS classification had some limitations. CMS was developed based on the gene expression in the tumor, irrespective of mutation status. However, mutation status was an essential factor to select the chemotherapeutic agent in current practice. Some CRC revealed intratumor heterogeneity. Multiple conflicting subtype assignments were sampled based on the tumoral region during tissue collection, using stromal‐based classifiers like CMS specifically when using biopsy samples.^16^ While clinical trial data was reanalyzed in accordance with CMS classification, further research will be necessary to translate the genetic data to clinical practice. Further investigation and analysis of CRC in accordance with CMSs will enable physicians to provide optimal, personalized treatment options and ultimately improve clinical outcomes for CRC patients.

## CONFLICT OF INTEREST

Authors declare no conflict of interests for this article.
